# Efficacy of sevoflurane as an adjuvant to propofol-based total intravenous anesthesia for attenuating secretions in ocular surgery

**DOI:** 10.1097/MD.0000000000006729

**Published:** 2017-04-28

**Authors:** Hou-Chuan Lai, Yun-Hsiang Chang, Ren-Chih Huang, Nan-Kai Hung, Chueng-He Lu, Jou-Hsiu Chen, Zhi-Fu Wu

**Affiliations:** a Department of Anesthesiology; b Department of Ophthalmology, Tri-Service General Hospital and National Defense Medical Center, Taipei, Taiwan, Republic of China.

**Keywords:** ocular surgery, propofol, secretions, sevoflurane

## Abstract

**Background:**

The incidence of nasal secretions into the operative field is as high as 5% in ophthalmic surgery under general anesthesia. It may induce postoperative endophthalmitis. Secretions under propofol-based total intravanous anesthesia (TIVA) are greater than sevoflurane anesthesia during surgery. Postoperative nausea and vomiting (PONV) after inhalational anesthesia is higher than TIVA and may increase intraocluar pressure. We investigated the effect of sevoflurane combination with propofol-based TIVA on nasopharyngeal secretions and PONV in ocular surgery.

**Methods:**

Fifty patients undergoing ocular operations were randomly assigned for propofol-based TIVA or propofol/sevoflurane anesthesia. In the TIVA group (n = 25), anesthesia was induced and maintained with propofol and fentanyl; in the propofol/sevoflurane group (n = 25), 1% sevoflurane anesthesia was added.

**Results:**

Nasopharyngeal excretion volume was significantly higher in the propofol-based TIVA group than in the propofol/sevoflurane group (31.0 ± 18.1 vs 13.7 ± 12.6 ml; *P* < .001). No significant difference in extubation time was noted (propofol-based TIVA: 6.4 ± 3.6 vs propofol/sevoflurane: 7.4 ± 3.0 minutes; *P* = .34). No postoperative endophthalmitis or PONV in both groups was observed.

**Conclusion:**

Sevoflurane attenuated secretions under propofol-based TIVA and did not increase the incidence of PONV or prolonged extubation in ocular surgery.

## 1 Introduction

The cause of postoperative endophthalmitis after ocular surgery is not known, and is probably variable. Several theories have been proposed. Rosenbaum^[1]^ suggested the possibility that endophthalmitis after strabismus surgery could have an endogenous origin. Good et al^[2]^ suggested that partial obstruction of the nasolacrimal duct and upper airway infection could be the risk factors for development of postoperative endophthalmitis in children undergoing cataract surgery. In addition, Naggle and Cooper^[3]^ reported that about 5% ophthalmic surgical cases under general anesthesia (GA) had secretions into the operative field. Besides, Bautista and Keech^[4]^ reported 2 cases under propofol anesthesia with excess secretions, resulting in surgical contamination in strabismus surgery. In our hospital, we also found that some patients had secretions toward the eye, resulting in contamination of surgical field in propofol-based total intravenous anesthesia (TIVA) during ophthalmic surgery,^[5]^ and it is more common in adults and in prolonged procedures.^[6]^ These may induce postoperative endophthalmitis; viridans streptococci most abundant in the mouth cause most cases of postintravitreal endophthalmitis.^[7]^ Therefore, anesthetics that produce less secretions are desirable. Previous studies also showed that propofol anesthesia would increase salivation.^[8–10]^ For this reason, TIVA is not suggested for ocular surgery. However, the incidence of postoperative nausea and vomiting (PONV) and the need for antiemetics were significantly less in the TIVA patients than in the inhalation anesthesia patients in ophthalmic surgery.^[11]^ Because PONV will increase intraocular pressure resulting in wound dehiscence and glaucoma.^[12,13]^ Therefore, TIVA is suitable for ocular surgery. In the literature, a rigorous comparison of the effects of propofol-based TIVA and propofol/sevoflurane anesthesia on secretions and PONV has not yet been performed. Therefore, in this study, we prospectively compared the effects of propofol-based TIVA and propofol/sevoflurane anesthesia on secretions and PONV in patients who underwent ocular surgery.

## 2 Methods

This study was approved by the Ethics Committee (TSGHIRB No: 2–104–05–129) of Tri-Service General Hospital, Taipei, Taiwan (Chairman, Professor Yu Mu Hsien) on 13th of October, 2015. All patients provided written informed consent before being enrolled. All methods were performed in accordance with the relevant guidelines and regulations by our IRB.

From October 2015 to March 2016, 50 patients in our medical center scheduled to undergo ocular surgery by 1 ophthalmologist under GA were enrolled in this study. Patients were randomized 1:1 into the propofol-based TIVA or propofol/sevoflurane anesthesia groups by using a table of random, computer-generated digits in sealed and numbered envelopes by an anesthesiologist. Participants and the ophthalmologist were blinded after assignment to interventions. Exclusion criteria were as follows: age <20 years or older than 80 years; American Society of Anesthesiologists (ASA) physical status of more than III; body mass index (BMI) >30 kg/m^2^; possible pregnancy; emergent surgeries; uremia; and liver disease.

All patients fasted overnight before surgery. To exclude the potential influence of diurnal variations of salivation, the patients were performed uniformly at the time around mid-day. There was no premedication before induction of anesthesia.

Regular monitoring, such as noninvasive arterial blood pressure, electrocardiography (lead II), pulse oximetry, and end-tidal carbon dioxide pressure (EtCO_2_) were applied in each patient. GA was induced with fentanyl, propofol, and rocuronium in all patients, then intubated and maintained with propofol or propofol/sevoflurane. All patients were monitored under bispectral index (BIS).

In the propofol-based TIVA group, anesthesia was induced using intravenous (IV) fentanyl (2 μg/kg) and 2% lidocaine (1.5 mg/kg). Continuous infusion of propofol was delivered subsequently using Schneider kinetic model of target-controlled infusion (TCI; Fresenius Orchestra Primea; Fresenius Kabi AG, Bad Homburg, Germany) with the effect-site concentration (*C*_e_) of 4.0 μg/mL. Rocuronium (0.6 mg/kg) was given when patients lost consciousness, followed by tracheal intubation.^[11,14–20]^ GA was maintained with TCI propofol infusion and 1.0 L/min flow with 50% oxygen. In the propofol/sevoflurane group, the anesthesia induction was as the TIVA group patients, whereas anesthesia was maintained using propofol infusion and 1% sevoflurane (inhaled concentration) with an oxygen flow of 1 mL/min. Repetitive bolus injections of rocuronium were prescribed as required throughout the procedure in both groups.

Maintenance of the *C*_e_ for the TIVA and propofol/sevoflurane was adjusted to keep BIS value between 40 and 60, and mean arterial blood pressure at 80 to 100 mm Hg. The EtCO_2_ pressure was maintained at 35 to 45 mm Hg. Once neuromuscular function returns, rocuronium (10 mg, IV) was administered as required. All patients received IV dexamethasone 5 mg for preventing PONV.

At the end of the procedure, propofol or sevoflurane was discontinued and the lungs were ventilated with 100% oxygen at a fresh gas flow of 6 L/min. Reversal of neuromuscular function was achieved by administrating neostigmine (0.03–0.04 mg/kg) with glycopyrrolate (0.006–0.008 mg/kg) once spontaneous breathing returned to prevent residual paralysis. When the patient regained consciousness by name with spontaneous and smooth respiration, the endotracheal tube was removed and the patient was sent to the postoperative anesthesia care unit for further care.

Total volume of secretions was determined by collecting them with frequent suction via nasal and oral cavities by using the suction apparatus (Mucus Extractor FG 14, Symphon, Taiwan) from the end of surgery to extubation of the endotracheal tube. Additionally, loss of consciousness (LOC) *C*_e_ of propofol, awakening *C*_e_ of propofol, maintenance *C*_e_, maintenance concentration of sevoflurane (%) and awakening concentration of sevoflurane (%), extubation time and the incidence of PONV within 24 hours after surgery was recorded.

Based on a preliminary data of 10 patients from our institution in the same surgical population, a power analysis was performed by reducing secretions as the primary variable. We calculated a sample size so that a reducing 15 mL (50%) of secretions would permit a 1-tailed type I error rate of α = 0.05 with a power of 80%. This analysis indicated that a sample size of at least 23 patients per group was necessary. To allow for potential dropouts, we enrolled a total of 25 patients in each group. Data are presented as the mean and standard deviation (SD) or number of patients. Demographic and perioperative variables were compared using Student *t* tests or Mann–Whitney *U* test, whereas the data were not normally distributed. Categorical variables were compared using chi-square test. Statistical significance was accepted for 2-tailed *P* values of <.05. The statistics was performed by using SigmaStat 3.5 for Windows.

## 3 Results

**Figure 1:**
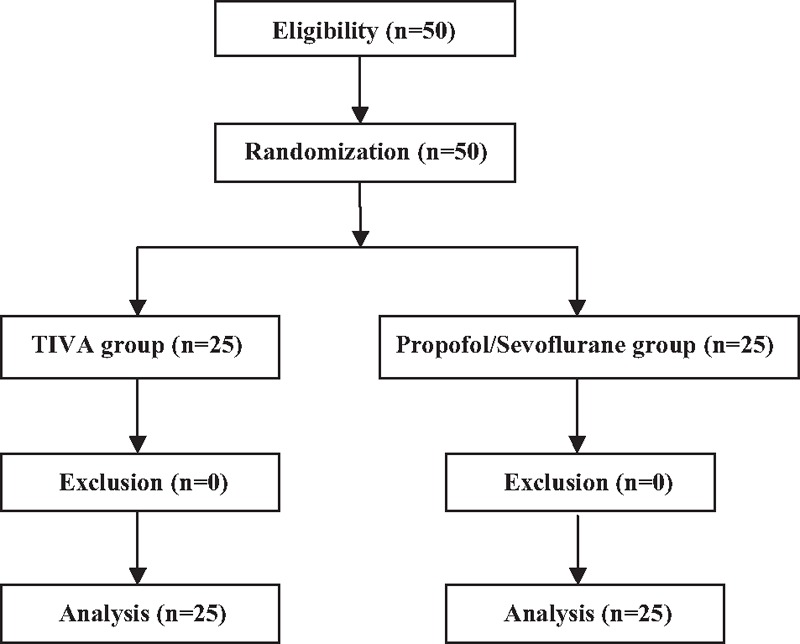
Flow diagram showing patient flow according to the study protocol.

Fifty patients undergoing elective ocular surgery under GA were performed successfully without being excluded. Ultimately, 50 patients completed the study: 25 in the TIVA group and 25 in the propofol/sevoflurane group (Fig. 1).

The groups showed similar patient characteristics (Table 1), and there was no significant difference between the groups in terms of operation time (TIVA group: 56.6 ± 24.4 vs propofol/sevoflurane group: 65.6 ± 32.4 minutes; *P* = .27) and anesthesia time (TIVA group: 87.3 ± 25.7 vs propofol/sevoflurane group: 97.5 ± 35.7 minutes; *P* = .25); extubation time (TIVA group: 6.4 ± 3.6 vs propofol/sevoflurane group: 7.4 ± 3.0 minutes; *P* = .34); and the LOC *C*_e_ (TIVA group: 3.2 ± 0.6 vs propofol/sevoflurane group: 3.4 ± 0.7 μg/mL; *P* = .21). Maintenance and awakening concentration of sevoflurane in the propofol/sevoflurane group was 0.7 and 0.03 ± 0.04%, respectively. The propofol awakening *C*_e_ values were 1.0 ± 0.3 and 0.7 ± 0.3 μg/mL in the TIVA and propofol/sevoflurane groups, respectively (*P* < .01). The maintenance propofol *C*_e_ values were 2.9 ± 0.5 and 2.2 ± 0.8 μg/mL in the TIVA and propofol/sevoflurane groups, respectively (*P* < .01). The volume of secretions was significantly higher in the propofol-based TIVA group than in the propofol/sevoflurane anesthesia group (31.0 ± 18.1 vs 13.7 ± 12.6 mL; *P* < .001).

**Table 1:**
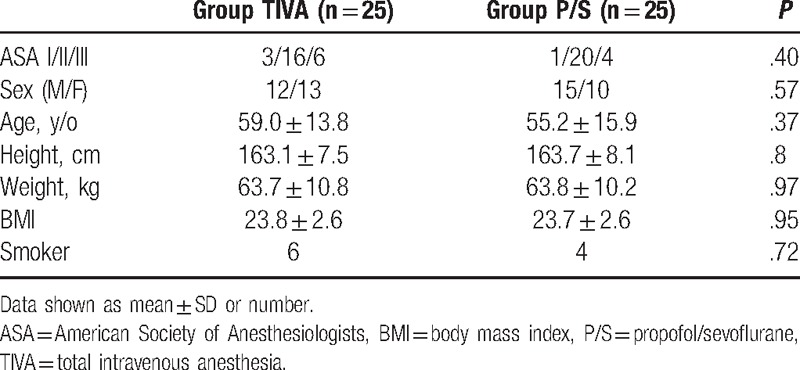
Patient characteristics.

Additionally, there was no patient with PONV within 24 hours and endophthalmitis within 2 weeks after surgery in both groups (Table 2).

**Table 2:**
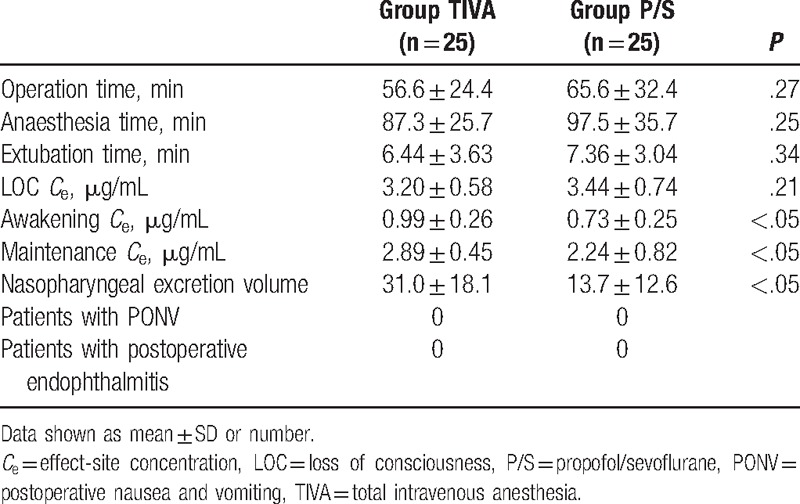
Comparison of perioperative characteristics and outcomes for the 2 groups.

## 4 Discussion

The major findings of this study revealed that combination with 1% sevoflurane anesthesia attenuates secretions under propofol-based TIVA in ocular surgery. For the purpose of preventing excess secretions resulting in surgical contamination and endophthalmitis in ophthalmic surgery, here, we emphasized not merely propofol combined with low concentration of sevoflurane, but caring the degree of neck flexion of the patients and test on the operating table to see if fluid leaving the nose could reach the conjunctival sac and draping the patient with a sealed plastic drape around the lower lids in ocular surgery under GA.^[3,6]^ If it happens, immediately staunching the flow of secretions before they reach the eye, removing the saturated drapes, reapplying sterile drapes, and povidone-iodine should be instilled into the nose as a part of the surgical field.^[3,6]^ In addition, we also found that propofol/sevoflurane anesthesia does not increase the incidence of PONV and prolonged extubation in ocular surgery, while TIVA combined with 1% sevoflurane under BIS monitoring.

The incidence of hypersalivation under propofol anesthesia in elective orthopedic and urological surgery was 60%,^[8]^ and in minor gynecological surgery was 25%.^[9]^ Whereas, propofol anesthesia compared with sevoflurane anesthesia would increase salivation in laryngeal microsurgery with unknown incidence.^[10]^ The mechanism of hypersalivation might be due to the fact that propofol increases the intracellular concentration of calcium and modulates the activation of P2X_4_ in submandibular acini.^[21]^ In contrast, Salukhe et al^[22]^ found that only 0.1% patients with hypersalivation in atrial fibrillation ablation under propofol sedation. In addition, Padda et al^[23]^ concluded that propofol compared with methohexitol anesthesia does not affect mucus secretion in the anesthetized dogs. Another study showed that propofol plus ketamine reduced salivary flow versus midazolam plus ketamine.^[24]^ Lahteenmaki et al^[25]^ directly compared propofol with isoflurane and found marked short-term hyposalivation in both groups. Moreover, Tsai et al^[26]^ reported that there was no significant difference in hypersalivation between TIVA (20.7%) and isoflurane anesthesia (20.9%) in soft tissue, oral, and orthopedic surgery for dogs. Furthermore, Agrawal et al^[27]^ concluded that hypersalivation might be due to stimulation of parasympathetic response from surgical site during surgery. To the best of our knowledge, the etiology of hypersalivation during anesthesia is due to anesthetics or surgical sites remains unclear, and needs further investigation.

We think that the effect of less nasopharyngeal excretions of propofol/sevoflurane group was due to sevoflurane reducing salivation compared with propofol. Kang et al^[10]^ showed that the lower concentration and secretion rate of chloride in the saliva of the sevoflurane group implies that salivary gland production was more inhibited in sevoflurane anesthesia. In addition, Kim et al reported that sevoflurane-induced decrease in airway secretion is due to the impairing chloride secretion indirectly by inhibiting the KCNQ1 channel in the tracheal epithelium and salivary gland.^[28,29]^ Also, it is supposed to be the mechanism of sevoflurane-induced decrease in secretions.

For attenuating secretions, an antisialagogue such as glycopyrrolate may be useful to reduce nasopharyngeal excretion, except in prolonged ophthalmic surgery.^[6,30]^ However, anticholinergics have cardiovascular adverse effects, including cardiac dysrhythmias and ischemia.^[31]^ Besides, anticholinergic agents may induce angle closure glaucoma due to ciliary muscle relaxation and pupil dilatation.^[32,33]^ For these reasons, the routine prescription of anticholinergics is not recommended, and anesthetics that produce less secretions are usually recommended, especially for ocular surgery.

Sevoflurane and propofol had similar efficacy for anesthesia; nevertheless, propofol-based TIVA may still be the preferred anesthetic because of its favorable anesthesia characteristics, such as high patient satisfaction and less frequent incidence of PONV.^[11,34–36]^ In this study, we found no patient with PONV in both groups. This finding may be resulting from all patients receiving IV dexamethasone 5 mg for preventing PONV; the patients received propofol/sevoflurane anesthesia; and the anesthetic technique was propofol-predominant and adjuvant sevoflurane.

The use of TIVA reduced the mean time to extubation by at least 9% compared with the use of inhalation anesthesia for ophthalmic surgery.^[11]^ However, our study showed no significant difference in extubation time between propofol-based TIVA and propofol/sevoflurane anesthesia. In the other previous studies, the extubation time was comparable between the propofol-based TIVA and inhaled sevoflurane groups.^[37,38]^ We concluded that our finding similar to above studies because of using BIS for monitoring anesthesia depth to achieve a BIS value between 40 and 60 in both groups and the like effect on extubation time between propofol and sevoflurane. In addition, there was no prolonged extubation in both groups.^[39]^

This study had 4 limitations, which should be considered. First, it was possible that objective functions of the salivary gland might have been different in the 2 study groups. Salivary gland secretion was a nerve-mediated reflex, and the volume of saliva secreted is dependent on the intensity and type of taste and on chemosensory, masticatory, or tactile stimulation.^[40]^ However, the risk of such a discrepancy between the 2 groups was minimal. In addition, all 50 patients received the same procedure, pars plana vitrectomy, and kept mean arterial blood pressure at 80 to 100 mm Hg during the procedure. A second limitation of the study was that we did not exclude smokers; smoking could, at least theoretically, increase secretions. However, smokers might not differ significantly from nonsmokers in salivary secretions.^[41]^ A third limitation was that our study was underpowered for PONV.^[42]^ We did not see any PONV in the 2 groups, and further investigation is needed. Finally, we did not distinguish the secretions from salivary gland or nasopharynx.

## 5 Conclusions

In conclusion, we showed that combination with 1% sevoflurane anesthesia attenuated propofol-induced excess excretions during ocular surgery. Besides, we found no prolonged extubation under BIS monitoring, no postoperative endophthalmitis, and no PONV in the 2 groups. It might suggest the clinical effect on the propofol/sevoflurane anesthesia.
